# Versatility of nasolabial flaps in oral cavity reconstructions

**DOI:** 10.4317/medoral.19376

**Published:** 2014-06-01

**Authors:** Estefanía Alonso-Rodríguez, José L. Cebrián-Carretero, María J. Morán-Soto, Miguel Burgueño-García

**Affiliations:** 1 Oral and Maxillofacial Surgeon, Hospital Universitario La Paz, Madrid, Spain; 2 Oral and Maxillofacial Surgeon, Head of Service. Hospital Universitario La Paz, Madrid, Spain

## Abstract

Objectives: Describe the techniques involved and the results obtained witn nasolabial flaps in small and medium-sized defects of the oral cavity. The procedure is an easy resconstructive option with a high success rate and with very good aesthetic and functional outcomes. Study Design: A retrospective analysis of 16 nasolabial flap reconstructions in 15 oncological patients with oral cavity defects undergoing single-stage surgical interventions. We evaluate the tumor type, its location, size, the resective and reconstructive techniques involved, as well as any complications. Results: Out of 15 patients, 9 were male and 6 female, with ages ranging from 60-85 years. The primary tumor was located in the mandibular or maxillary gingiva in 7 patients, the lateral margin of the tongue in 5, the floor of the mouth in 3 and the mandibular symphysis in a single patient. The tumors were of a small to medium size. All patients underwent intraoral resections. In most cases, a cervical dissection was performed. All flaps were completed as single-stage surgical interventions, with 14 unilateral and 2 bilateral procedures. Five patients had received radiotherapy treatment for previous tumors. During the follow up period, which ranged from 4 months to 8 years, only one patient required their flap to be thinned, there were two incidents of surgical wound dehiscence, two hematomas and one orocutaneous fistula, none of which affected the survival of the flap. Conclusions: The nasolabial flap proves highly versatile in oral cavity reconstructions, coupled with a minimal morbidity of the donor region and good aesthetic and functional results. Its high vascularity allows for cervical dissections to be carried out or even for radiotherapy to be administered prior to it. It is straightforward, safe, and carrying it out as a single-stage intervention makes it the ideal surgical option for small to medium intraoral defects in edentulous patients with other comorbidities.

** Key words:**Nasolabial flap, oral cavity reconstruction, oral cavity defects.

## Introduction

The oncological sequelae affecting the oral cavity lead to significant functional and aesthetic changes. When the defect is of a more complex nature, microvascular free tissue transfers tend to be the treament of choice for the reconstruction. However, in the case of small or medium-sized oral cavity defects, the use of nasolabial flaps facilitates a fast and simple procedure, coupled with a very high success rate.

The nasolabial flap was first described in the Sushruta Samhita text in 600AD as a means of correcting cutaneous defects. In 1868, Thiersch was the first to employ this technique to perform intraoral reconstructions of palatal fistulas. Since then, various modifications have been described, including full thickness (1) and musculocutaneous flaps (2), together with the subcutaneous approach, which is currently the most widely used. In this way, nasolabial flaps have come to be considered a safe and useful surgical option in the reconstruction of oral cavity defects. The procedure can be performed as a single-stage surgical intervention with primary

closure of the donor región (3) or in two stages if the pedicle dissection is deferred (4-6).

We present a retrospective analysis of 16 nasolabial flap reconstructions in 15 oncological patients with oral cavity defects. The flaps were carried out in the supramuscular plane and as a single-stage intervention, providing satisfactory results without the need for a second surgical intervention. This procedure is ideal for more elderly patients with systemic disorders, amongst whom we can avoid a higher morbidity.

## Material and Methods

Between 2004 and 2012, fourteen patients diagnosed with epidermoid carcinoma of the oral cavity and one patient treated for a mandibular sarcomatous carcinoma underwent surgical reconstruction with 16 nasolabial flaps in the Department of Oral and Maxillofacial Surgery of the Hospital Universitario La Paz. We carried out a retrospective analysis evaluating the data relating to the tumor type, its size and location, together with the resective and reconstructive surgical techniques employed in managing oncological patients.

The design of the flap includes a superior margin that is situated more than 5-7 mm away from the medial canthus to avoid ectropion, a medial border that runs along the length of the nasolabial fold, an inferior border parallel to the lower dental arch and a lateral border that is dependent upon the size of the defect that needs to be covered. A supramuscular plane of dissection is used. Once dissected, a transbuccal tunnel posterior to the orbicularis muscle is created. The tunnel must be wide enough to transfer the flap into the intraoral cavity without constricting itself. During a single-stage surgical intervention, the area resting within the tunnel is de-epithelialized and, after suturing the intraoral flap in its final, the donor region is closed recreating the facial folds.

## Results

Out of 15 patients, 9 were male and 6 female, with a mean age of 73 years (range 60-85 years).

A total of 16 intraoral reconstructions were performed due to one patient undergoing two entirely separate single-stage nasolabial flap reconstructions for two independent tumor lesions.

The primary tumor was located in the mandibular or maxillary gingiva in 7 patients, the lateral margin of the tongue or glosso-mandibular sulcus in 5, the floor of the mouth in 3 and the mandibular symphysis in a single patient. The largest defects required a bilateral nasolabial

flap reconstruction and none of the small to medium-sized lesions exceeded 5 cm in diameter ( Table 1).

**Table 1:**
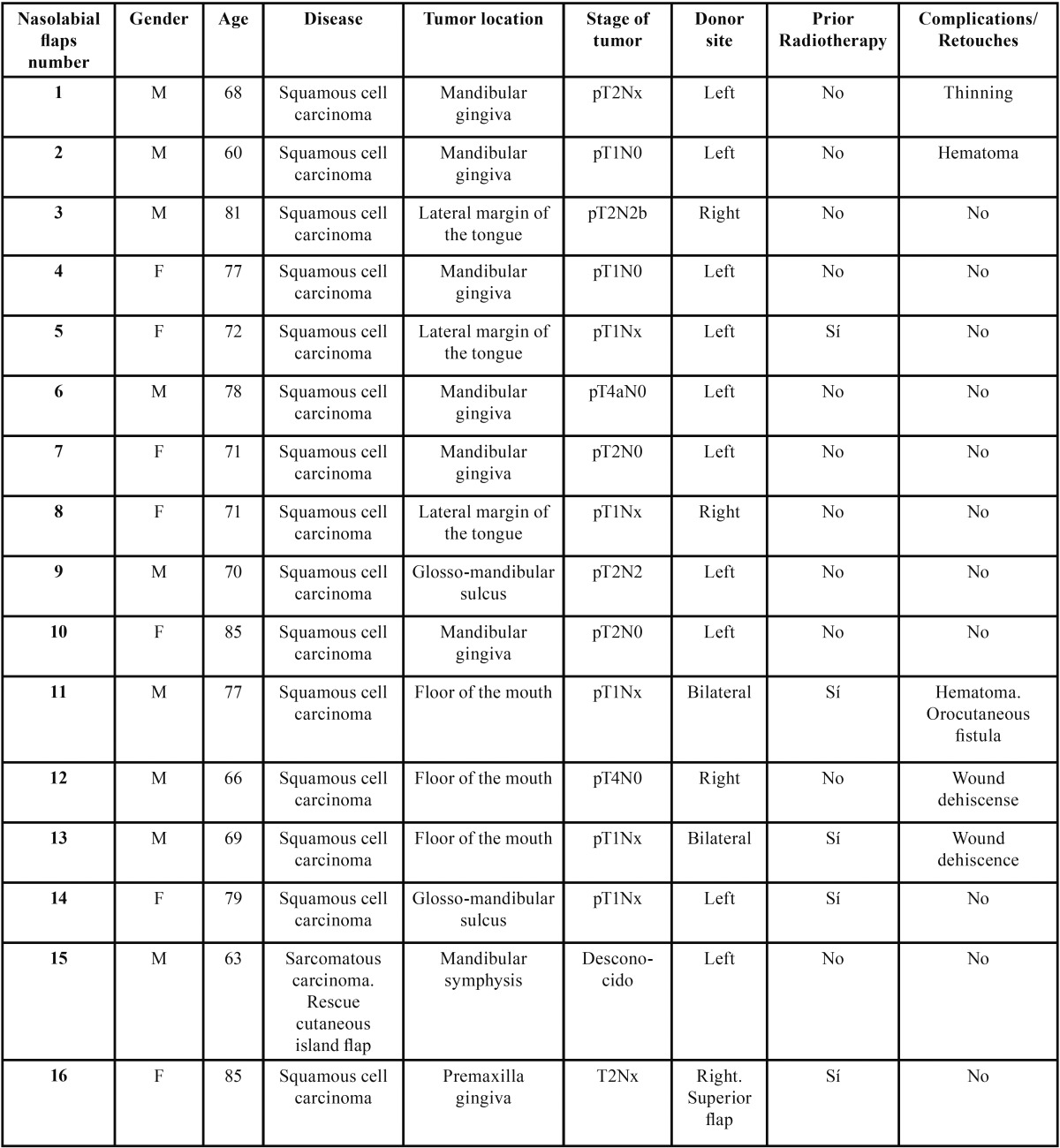
Dates of nasolabial flaps used in the reconstruction of intraoral cavity defects in oncological patients.

All of the patients underwent intraoral tumor resections. Those with epidermoid carcinomas of the mandibular or maxillary gingiva had their gingiva resected, together with the alveolar bone and adjacent teeth, the oral vestibule and floor of the mouth or a portion of the hard palate. In the case of lingual tumors, the floor of the mouth was resected in addition to the partial glossectomy. Cervical dissections were carried out in almost all procedures and were in line with our established treatment protocols. Five patients had received radiotherapy treatment for previous tumors. All of the nasolabial flaps were completed as single-stage surgical interventions (14 unilateral and 2 bilateral); all utilized inferior pedicle flaps except for a single case based on a superior flap. The latter one was an epidermoid carcinoma located in the maxillary gingiva and its defect was reconstructed using a right nasolabial flap based on a superior pedicle. The tecnique is the same as that of an inferior based pedicle, except that the flap is transferred into the intraoral cavity with a transbuccal tunnel lateral to the level of the alar base.

The patient diagnosed with a sarcomatous carcinoma attended our clinic following a failed reconstructive attempt with peroneal and local flaps post resection. The defect was then reconstructed using an antebrachial flap and a new microvascularized peroneal flap. After several episodes of venous congestion and massive cervical bleeds, the cutaneous peroneal island flap suffered a partial necrosis with bone exposition. It was subsequently reconstructed utilizing a successful nasolabial flap design.

Over the course of the follow up period, which ranged from 4 months to 8 years, the only complications encountered were two incidents of surgical wound dehiscense, two hematomas and one orocutaneous fistula. The patient who was affected by both the fistula and one of the hematomas was a known hypertensive with COPD and a heavy smoker. These complications were resolved with minor surgical interventions and had no repercussions on the survival of the flap. Only one patient required their flap to be thinned. We now present two case reports.

-CASE 1

An 81-year old man, ex-smoker of 20 years and with a past medical history of renal and bladder cancer. He attends the clinic due to an ulcerative lesion measuring 2.5 x 2 cm in diameter. It has been present for several months and is located in the mucosa of his right glosso-mandibular sulcus and right lateral lingual border. The decision is made to perform a block resection of the floor of the mouth and right glosso-mandibular sulcus under a general anesthetic in addition to a right partial glossectomy with periostomy of the mandibular lingual aspect. All the molars in the fourth quadrant were absent and, for this reason, pieces 44 and 45 were extracted to facilitate the reconstruction.

**Figure 1:**
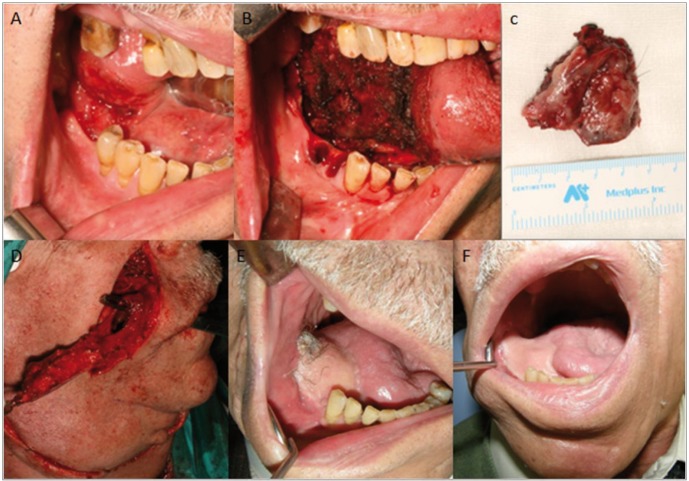
A) Ulcerative lesion in right glosso-mandibular sulcus and right lateral lingual border. B) Intraoperative image after intraoral resection of the lesion with wide margins. C) Surgical specimen. D) Intraoperative image showing nasolabial flap design. Transbuccal tunnel allows the flap to be transferred into the intraoral cavity. E, F) Postoperative images before and after radiotherapy treatment.

Though he did not present with any suspicious cervical adenopathies, we followed our protocols and carried out a right suprao-mohyoid dissection. The intraoral defect was reconstructed with a right nasolabial flap that was tunneled, de-epithelialized and sutured in its final position as a single-stage procedure. All traces of intraoral hair disappeared following the patient’s postoperative radiotherapy, which resulted in a satisfactory aesthetic outcome (Fig. 1). At present the patient is still being followed up and shows no signs of relapse.

-CASE 2

**Figure 2:**
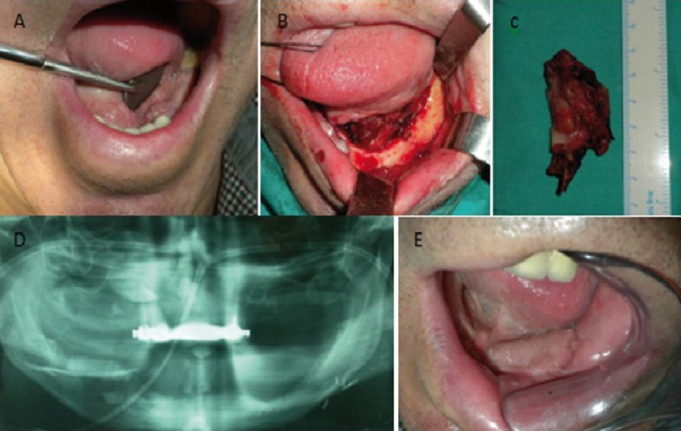
A) Clinical image showing a lesion in the alveolar gingival of third quadrant. B) Intraoperative image showing the intraoral defect after resection. C) Surgical specimen. D,E) Orthopantomography and clinical image, 4 months after surgery.

**Figure 3:**
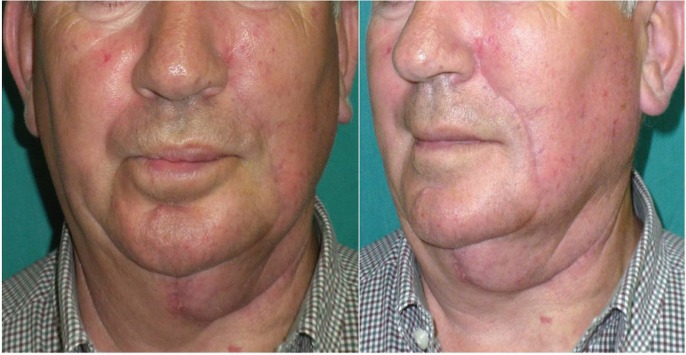
Second case pictures, with side and front view, showing a satisfactory aesthetic outcome, one month after surgery.

A 60-year old man, diabetic, with atrial fibrilation, current smoker and drinker, presents with an epidermoid carcinoma measuring 2 cm in diameter, located in the alveolar gingiva of the third quadrant, distal to a fixed prosthesis and extending into the floor of the mouth. The patient was found to be edentulous posteriorly and with radicular remains. No palpable cervical adenopathy. A marginal mandibulectomy sacrificing the left inferior alveolar nerve was performed, together with an extraction of the the radicular remains, a left supraomohyoid

dissection and left nasolabial flap as per the technique previously described. The patient has a good aesthetic outcome (Figs. 2,3) and continues to be followed up without any signs of disease recurrence.

## Discussion

The nasolabial flap has traditionally been described as an axial flap that depends on the angular, infraorbital, transverse facial and dorsal nasal arteries of the face (7). However, the vast number of anastomoses and the rich subdermal vascular plexus also mean that it can be

utilized as a random skin flap (8). It is possible to create flaps based on a medial or lateral pedicle; an inferior pedicle, useful in the reconstruction of labial and oral cavity defects; or based on a superior pedicle, useful for defects affecting the tip or ala of the nose, cheek or lower eyelids (9,10). As described in this article, nasolabial flaps can also be used to address intraoral defects affecting the maxillary gingiva, the palate or the buccal mucosa. Lazaridis et al. describe a study in which 9 patients undergo single-stage surgical interventions for the reconstruction of intraoral defects with nasolabial flaps, four of them involving a superior pedicle. In addition to a good aesthetic outcome, the incidence of postsurgical trismus is reduced thanks to the proximity of the donor region during the reconstruction, enabling a primary closure with little tension (11).

The versatility of the nasolabial flap in the reconstruction of oral cavity defects is widely known and accepted. It is capable of providing sufficient tissue to adequately reconstruct small or medium-sized defects of the floor of the mouth with the option of performing a bilateralprocedure when the defect is deemed to be substantial (12). The largest defects required reconstructions with bilateral nasolabial flaps, though the tumors were small to medium in size and never exceeded 5 cm in diame-ter. A unilateral flap can cover a defect of around 3 cm, whilst a bilateral one can cover defects of up to 5-7 cm in length (4,6,9,12).

Patients with comorbidities can benefit from reconstructions utilizing this local flap and avoid the longer surgical times involved in microvascularized flaps. It is possible to perform this reconstruction with a nasolabial flap as a two-stage surgical intervention. The procedure is similar that of the single-stage intervention described but, after tunnelling the pedicle and suturing it intraorally, a further 1 to 3 weeks must elapse before the pedicle can then be dissected (4-6). We prefer to de-epithelialize the base of the flap, bringing about the primary closure of the donor region and omitting the need for a second intervention, thus avoiding further surgery, its associated cost and a greater morbidity for the patient. In dentulous patients it is possible to block the bite to prevent the pedicle from being damaged. However, this technique is ideal for subjects who are edentulous in the ipsilateral canine and premolar regions, as their chances of prosthetic rehabilitation with muco or implant-supported prostheses is possible even during a single-stage surgical intervention. Another option is to carry out a musculocutaneous nasolabial flap (2), dissecting the major and minor zygomatic muscles and the levator labii superioris muscle, thus ensuring a vascular supply. However, the morbity of the donor region is greater with facial weakness and a poor aesthetic outcome. The vascular supply as a random tissue flap is also high and carries a lower morbidity.

The high vascularity of the flap enables a uni or bilateral ganglion dissection with facial artery ligation without having a detri-mental effect on the viability of the flap (13-15). Most patients, in line with our treatment protocols, underwent elective neck dissection without it having a negative effect on the flap.

The patients who have received radiotherapy treatment are at no greater risk of complications, not only due to the flap’s excellent vascularity (14), but also because most of the donor region is outwith the normal field of irradiation. Out of the 5 patients in our series who had previously received radiotherapy treatment for other tumors, two suffered complications. These included the dehiscence of a surgical wound and a hematoma, in addition to an orocutaneous fistula in the same patient. Both of the patients affected were also heavy smokers and the complications had no repercussion on the survival of the flap.

In most of our patients the aesthetic and functional outcomes were very good. The presence of intraoral hair amongst males disappears with postsurgical radiotherapy treatment or with further depilation techniques. The aesthetic repercussions are minimal and the functions of phonation, deglution and mastication are preserved in a satisfactory manner. There are studies which suggest that long-term postsurgical sensory reinnervation may even be possible with this and other types of oral cavity flaps (16-18).

Generally speaking, the complication rate is low. In our series of 16 flaps, 4 suffered from complications but all of them were resolved with minor surgical procedures and medical treatment without any of the flaps showing signs of necrosis. This represents a 100% survival rate, which is comparable to the results published from other series. El-Marakby et al in 2012, in a series of 20 patients undergoing single-stage nasolabial flap reconstructions also reported a 100% survival rate (3). The study by Varghese et al in 2001, with 224 patients, represents the longest series of nasolabial flaps with intraoral reconstructions carried out as as a two-stage surgical intervention. They reported a partial necrosis rate of 5.5% and a complete one of 6.3% (6).

## Conclusion

The nasolabial flap proves highly versatile in oral cavity reconstructions in oncological patients, coupled with a minimal morbidity of the donor region and good aesthetic and functional results. It is easy to carry out and has a high success rate, making it the ideal surgical option for small to medium intraoral defects in edentulous patients with other comorbidities.
